# Does having a pet influence the physical activity of their young female owners?

**DOI:** 10.1186/s12889-019-7962-z

**Published:** 2019-12-12

**Authors:** Kristýna Machová, Klára Daďová, Helena Chaloupková, Ivona Svobodová

**Affiliations:** 1 Department of Ethology and Companion Animals, Czech University of Life Sciences, Prague, Czech Republic; 2 Department of Adapted Physical Education and Sports Medicine, Faculty of Physical Education and Sport, Charles University, J. Martího 31, 162 52 Prague, Czech Republic

**Keywords:** Pet ownership, Human-animal interaction, Exercise, Dog walking, Horse riding

## Abstract

**Background:**

Many studies have shown that having a dog has an impact on the increase in physical activity (PA) of people. However, what is often not taken into account in many such studies is owning of other pets. The aim of this study was to compare PA levels between animal owners and non-owners and to research potential differences between owners of different kinds of animals.

**Method:**

111 young females of mean age 21 ± 1.2 years enrolled in this cross-sectional study. Czech version of short International physical activity questionnaire (IPAQ) was used to assess PA level, supplemented with a question about whether they owned an animal and what kind.

**Results:**

People who owned a pet had higher frequency and duration of moderate physical activity (MPA) and spent more MET/min/wk. (*p* < 0.05). This difference has projected into total PA duration and also into calories burned in a week. Furthermore, a statistically significant difference between subgroups of animal owners was also confirmed for MPA and total PA in favour of horse owners.

**Conclusions:**

Animal owners generally reported higher PA levels compared to people who do not own any pets. However, similarly significant in this particular age group was the kind of animal these young women owned.

## Background

Several past studies have clearly demonstrated a direct link between health and the extent of regular physical activity (PA). Therefore, people who want to be healthy should aim to exercise regularly, intensively and sufficiently on a lifelong basis [1, 2]. The World Health Organization (WHO) recommendation for adults aged 18–64 years is to do at least 150 min of moderate-intensity physical activity or at least 75 min of vigorous-intensity physical activity per week. However, about 23% of adults aged 18+ (20% for men and 27% for women) fail to meet this recommendation [3].

It is well known that there is a gradual reduction of PA between adolescent years and early adulthood [4]. The extent of PA is also related to many other factors at this age [5]. As the proportion of intellectual work continues to increase, many people find their PA reducing; eventually, they lose their physical fitness [6]. The different intensity of PA during adolescence compared to other phases in life suggests that late adolescence and early adulthood may be critical periods regarding PA. Many researchers have found that a greater percentage of adolescent males report being highly active compared to adolescent females, who report more frequent sedentary behaviour [7, 8]. There is compelling evidence that young females experience varied challenges that sometimes impede their sustained engagement in physical activity [9]. Becoming a parent could be one of the factors which influence the levels of PA in young women in particular [10]. Moreover, parents (especially mothers) have high impact on future lifestyles of their children as well as on their children’s attitude to PA [11]. For this reason, it is important for women to adopt healthy behaviour in young age.

It turns out that finding motivation to go for a walk or doing other forms of moderate intensity exercise at this age is crucial in preventing decrease in PA in future stages of life [12]. One of many reasons for deciding to purchase a pet (e.g. a dog) is the intention to ensure a long-term motivation for regular PA. In this case a pet serves as companion; it is an animal kept primarily for a person’s company or entertainment, rather than being a working animal, livestock or a laboratory animal. Number of studies have shown a positive influence of dog-ownership on human PA [13–18] as well as on the extent of movement through dog-walking [19]. In addition to dogs, keeping horses, whether for sport or as pets, turns out to be another way of increasing PA [20]. In contrast, there are also people who take care of many other kinds of animals and the influence of these animals on the PA level of their carers is mostly unknown.

The aim of this study was to investigate how owning a pet affects the PA of young female adults, with the focus on dogs, horses and other domestic animals and to compare their physical activity to women of same age who do not own any animals.

## Methods

### Participants

There were 111 female participants (21.14 ± 1.24 years; mean ± SD) enrolled in this study. Eligible were young women of age 18 to 25 years, having gone through at least 13 years of school education. We specifically chose young women for this study as they are in higher risk of PA decline [3, 21].

Participants were recruited in 12 different locations of the Czech Republic. They were randomly contacted through Czech University of Life Sciences students and their participation was conditioned with their consent to be part of the study. They were informed that they would not be receiving any financial or nonfinancial compensation for their participation. Questionnaires were distributed throughout the autumns of 2017 and 2018.

We received oral approval from all participants that they agreed to participate in this study; all data were anonymous. Students of the Czech University of Life Sciences have been instructed in how to use IPAQ (International Physical Activity Questionnaire) and have been trained to be able to answer any questions in this questionnaire in a clear and effective manner. Questionnaires with incomplete data were excluded.

### Procedures

The IPAQ was administered to summarize the levels of intense and moderate PA, walking time and sedentary time in the last 7 days [22]. The evaluation of the levels of PA was carried out by applying the short form of the questionnaire in Czech language (Center for kinanthropology research, 2006) [23]. The respondents were asked to report the number of days and the duration of vigorous physical activity (VPA), moderate physical activity (MPA) and walking physical activity (WPA). This short version has demonstrated an acceptable test-retest reliability and criterion-related validity in a 12-country evaluation study [22].

All scores were expressed in MET-minutes/week (www.ipaq.ki.se). For the analysis of IPAQ data IPAQ guidelines were followed [24].

The short form was chosen for being less time-consuming and for containing all the monitored data required for our test. As well as asking participants whether they owned an animal and what kind it was, the questionnaire also asked for demographic data such as gender, age and the number of years of education. In order to calculate the Body Mass Index (BMI), the questionnaire also asked for the participants’ height and weight.

For analysis, subjects were divided into two groups: animal owners (AO) and non-animal owners (NAO). The animal owners group was further divided into the following subgroups: 1. dog only or dog and other small animal owners /i.e. excluding horses/ (DO), 2. horse only or horse plus any other animal owners /i.e. including dogs/ (HO), 3. all other animals owners, no matter what kind /cats, turtles, snakes, mice, birds/ (OAO). We created these groups because many participants owned different combinations of animals and if we focused only on single animal owners, we would not have enough data to analyse.

### Statistical analysis

All data were analysed using STATISTICA (StatSoft, Tulsa, USA, version Cz. 7). As most of the data did not meet standard criteria of normality, the differences between AO and NAO in VPA, MPA and WPA including related variables were evaluated using non-parametric Mann-Whitney U test. The difference between specific animal owner groups was evaluated by Kruskal-Wallis ANOVA including post-hoc analyses. Results were considered statistically significant when *p* ≤ 0.05.

## Results

Table 1 shows demographic data of animal owners (AO) and non-animal owners (NAO). The age in the two groups did not differ significantly, neither did their body heights or years of education. However, there was a significant difference between the two groups regarding body weight and body mass index where the animal owners showed lower BMI.

**Table 1:** Demographic data of animal owners (AO) and non-animal owners (NAO)

	AO (*n* = 60)	NAO (*n* = 51)	*p*-value
Mean age ± SD (yrs)	21.23 ± 0.98	21.02 ± 1.49	0.170
Mean weight ± SD (kg)	60.39 ± 7.46	63.39 ± 6.80	< 0.001⁎
Mean height ± SD (m)	167.97 ± 6.98	168.69 ± 2.87	0.839
Mean BMI ± SD (kg.m^−1^)	21.41 ± 2.39	22.25 ± 1.99	0.021⁎
Mean length of education	14.25 ± 1.10	14.35 ± 1.31	0.880

*p* ≤ 0.05

Table 2 compares AO and NAO using data from IPAQ (International Physical Activity Questionnaire). The main difference between the two groups showed in the number of days the participants spent on MPA (i.e. frequency of PA) and in the total amount of minutes they spent on MPA a day (i.e. duration of PA) with corresponding difference in MET-min a week in moderate activity level. The number of total minutes in PA (sum of VPA, MPA and WPA) was significantly different between AO and NAO (*p* = 0.02) in favour of AO, as well as total MET-min a week. Consequently, there was also a statistically significant difference between the groups in calories burned per week (*p* = 0.01). On the other hand, there was not a statistically significant difference in variables describing frequency and duration in VPA and WPA.

**Table 2:** Comparing the AO and NAO using data from IPAQ (International Physical Activity Questionnaire), median (IQR)

	AO (*n* = 60)	NAO (*n* = 51)	*p*-value
Number of days with VPA	2 (3.5)	2 (3)	0.622
VPA (min) / week	77.5 (105)	60 (70)	0.179
VPA (MET-min) / week	1920 (3840)	1080 (2400)	0.121
Number of days with MPA	3 (2.5)	2 (3)	0.003⁎
MPA (min) / week	60 (150)	60 (90)	0.019⁎
MPA (MET-min) / week	900 (2280)	320 (960)	0.004⁎
Number of days with WPA	7 (0.5)	7 (2)	0.546
WPA (min) / week	120 (120)	120 (120)	0.456
WPA (MET-min) / week	2772 (2772)	2376 (2772)	0.344
Total PA (MET-min) / week	6212 (4772)	3990 (3363)	0.005⁎
Calories / week	6391.6 (5169)	4065 (3654)	0.011⁎
Total PA (min) / week	294 (240)	210 (180)	0.016⁎

*p* ≤ 0.05

Table 3 shows comparison of the above-mentioned variables between NAO and different groups of animal owners (dog owners – DO, horse owners – HO, and other animal owners – OAO, see the method section).

**Table 3:** Reported PA in NAO and subgroups of AO including multiple owner group, median (IQR), df = 3, *n* = 111

	NAO (*n* = 51)	DO (*n* = 31)	HO (*n* = 18)	OAO (*n* = 11)	H	*p*-value
Number of days with VPA	2 (3)	2 (5)	3 (2)	2 (4)	0.95	0.812
VPA (min) / week	60 (70)	60 (120)	120 (90)	90 (180)	4.33	0.228
VPA (MET-min) / week	1080 (2400)	1440 (2880)	1920 (3360)	2880 (5760)	4.28	0.232
Number of days with MPA	2 (3)	3 (3)	3.5 (3)	3 (2)	11.14	0.011⁎^b^
MPA (min) / week	60 (90)	40 (75)	180 (90)	120 (160)	18.41	0.0004⁎^b,d^
MPA (MET-min) / week	320 (960)	480 (1120)	2160 (2640)	1440 (2000)	17.20	0.0006⁎^b,d^
Number of days with WPA	7 (2)	7 (1)	7 (0)	7 (2)	0.79	0.851
WPA (min) / week	120 (120)	120 (120)	150 (120)	150 (120)	0.67	0.880
WPA (MET-min) / week	2376 (2772)	2772 (2772)	3168 (2772)	2970 (2475)	1.15	0.766
Total PA (MET-min) / week	3990 (3363)	5199 (4506)	6945 (2899)	6558 (7806)	13.52	0.004⁎^b^
Calories / week	4065 (3654)	5409 (4633)	6945 (3534)	6776 (6537)	9.96	0.019⁎^b^
Total PA (min) / week	210 (180)	260 (190)	345 (120)	420 (318)	13.84	0.003⁎^b,d^

*n* number of subjects, *IQR* interquartile range

^a^ NAO and DO

^b^ NAO and HO

^c^ NAO and OAO

^d^ DO and HO

^e^ DO and OAO

^f^ HO and OAO

*p* ≤ 0.05

**Fig. 1:**
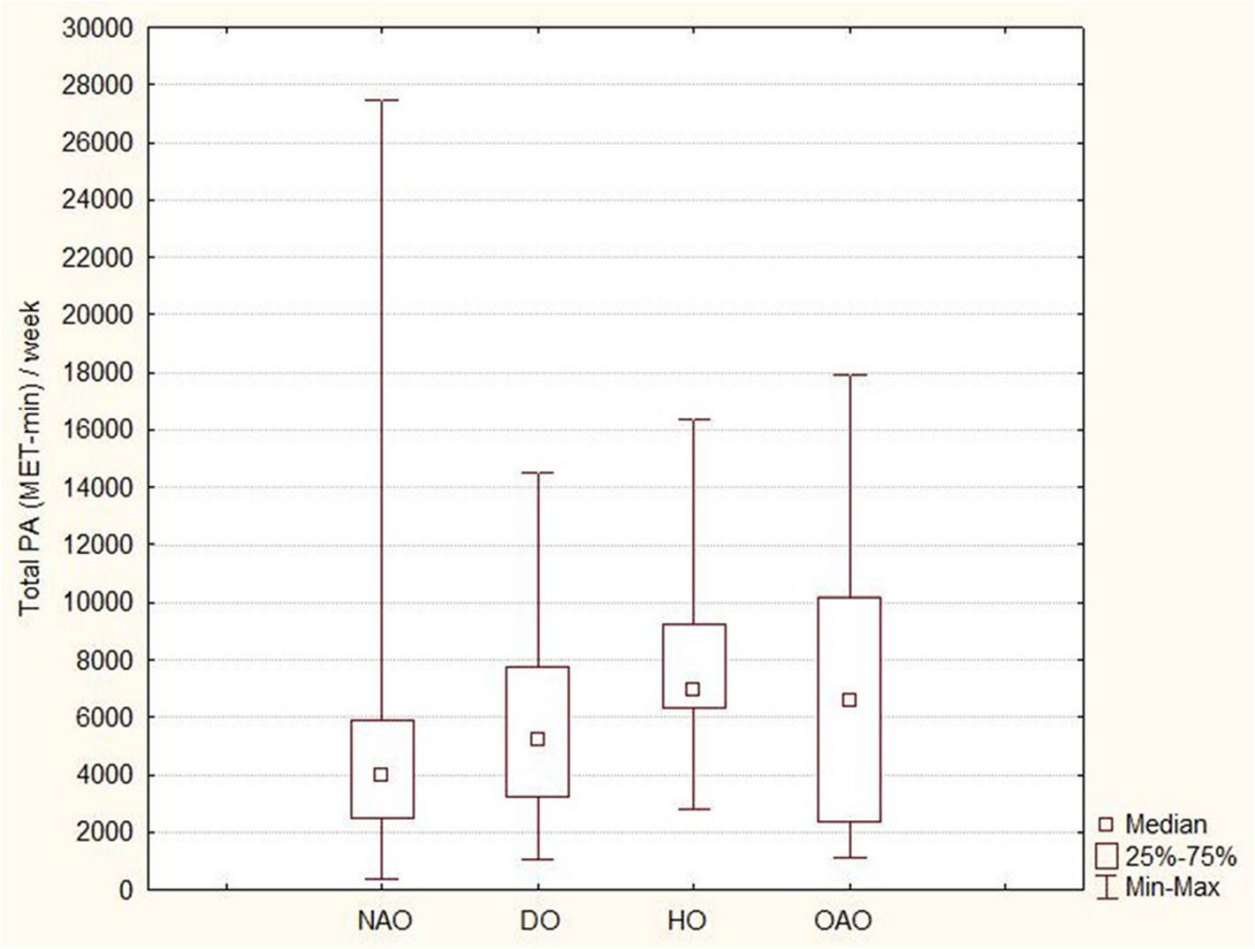
Comparison of total PA expressed as MET-min a week between non-animal owners (NAO), dog owners (DO), horse owners (HO) and other animal owners (OAO)

Statistically significant difference between the groups of animal owners was confirmed for the same variables in MPA (i.e. moderate intensity) and total calories, total MET-min (see Fig. 1) a week and total time spent with PA. The post-hoc analysis showed that in all above-mentioned variables the horse owners had significantly higher MPA then the non-animal owners. Furthermore, the horse owners had significantly higher duration of MPA and significantly higher total PA in a week (mins) than the dog owners. However, a great interindividual variability existed in the data.

## Discussion

Our results show that, as described in other studies, possible positive influence of animal ownership on the PA level could be expected [1, 17, 25–31]. Animal owners, as whole group, showed in comparison with the non-owner group, higher level of moderate PA (MPA). This result was also visible in the total calories burned and total MET-min/wk. What seems quite interesting is that dog owners (DO) did not show significantly higher walking activity compared to non-animal owners or owners of other animals [14, 16, 19].

Very interesting and innovative is grouping animal owners according to animal kind and comparing of reported PA within those groups. In this case, an unexpected result was that the dog owners (i.e. those who only have a dog or those who have a dog as well as other small animal, see our grouping above) have the lowest MPA (min/wk) out of all the AO groups. This is contrary to the study of Brown and Rhodes [32], who, compared to our research, reported higher MPA and walking activities in the dog owners. In contrast to that, reported PA in our DO group is similar to PA of the non-owner group. Moreover, the PA is significantly lower than in horse owners. This probably signifies that if people own a dog it does not necessarily mean that they walk it frequently. Similar problem had been published in a study of Westgard et al. [2]. Even-though people had a dog, some of them did not actually walk it, so their PA became stagnant. As a result, the very fact of owning an animal sometimes does not provide enough motivation to increase PA, as suggested also by Higgins et al. [30]. However, the data from previous studies are not consistent. In study of Cutt et al. 23% of dog owners did not walk with their dog [33], in study of Richards et al. up to 70% of dog owners did not walk their dog enough to achieve health benefits [19]. For example, study of Yabroff et al. [34] showed that the dog owners were less likely to use walk as means of transportation but were more likely to walk for leisure than the non-dog owners.

Another interesting finding is that many people own more than one animal – i.e. as the groups of single animal owners were rather small, about half of the AO sample consisted of people who owned more than one animal. Thus, it was rather difficult to divide respondents according to pet types in a reasonable way. For example, 52% of dog owners also owned another small animal. HO group members usually owned also a dog (83%) and 61% of horse owners owned more than 3 animals. This fact could cause a mixed effect and distort the results. Nevertheless, it seems that the people who own a horse report the highest values of PA. This could be expected as there is a strong justified presumption that horse owners are going to be very motivated to ride their horses and thus produce PA as a result of high cost of acquisition and ownership.

Our findings may explain inconsistencies in some previous studies as it is showing that their results could have been distorted by the fact that they owned other animals including horses, which according to our study could have significant influence on PA level.

The highest physical activity was reported by horse owners (HO) both in duration and frequency of MPA and in total PA. This result is in an agreement with the study of Sjogren et al. [10], where dog and horse ownership were one of the strongest factors for participation in outdoor recreational PA. We could speculate that there might be higher personal motivation in this group which is a key factor for PA level increase as reported by Lim and Rhodes [35].

If participants in our study owned 3 or more animals (including dogs and horses), they reported the highest level of MPA in minutes, MET-min a week and total PA time (data from this grouping are not shown). It seems that owners of dogs, horses and other domestic animals are probably the most active people, due to carrying out a lot of physical exercise with them.

The question whether more active people own animals or whether animals make people more active is not easy to answer. Unfortunately, it cannot be determined with this type of study design, but we assume animal ownership can bring many positive effects to people, both physical and psychosocial, and thus contribute to improvement in public health.

According to research data, girls show less physical activity than boys because of fear of not being good enough to participate. Particularly making an error could result in possible public embarrassment thereby diminishing social standing. Participating in individual sport activities was reported to less stressful than doing team sports because there was no added pressure of letting potential team members down by not performing well [9]. This leads us to conclusion that owning an animal seems to be a possible way to help motivate young females to increase their PA, as the fear of failing would not apply here.

Being motivated to exercise is essential in order to maintain adequate level of physical activity. In order to support this motivation, it is necessary to understand it well in the context of particular cultural environment and specific age group so that it can be targeted effectively. The positive effect of animal ownership on PA has been proved. It is possible to use the positive effect animal ownership has on PA of people and make it a good habit from childhood with the aim to transfer this into adulthood. This would be a way to influence young women who are at more risk of reducing physical activity than are men of the same age.

Given the potential that animal ownership has on increasing the levels of PA across the population, the near future urban planning should support physical activities for animal owners and their pets. Also, population-levels of PA could be increased thanks to growing popularity of companion animals who often become member of family [36].

### Limitations of the study

We need to point out a great variability of the data so the results should be interpreted carefully. On the other hand, high variability of data from IPAQ is quite common, as can be seen for example in a study carried out by Sklempe et al. [37].

Another limit of our study is that participants were not a representative sample and it was not possible to examine potential moderating factors. One of this possible factors was that we did not know whether the health conditions of all the subjects was roughly the same, or whether they had some health problems that might prevented them from exercising with their pets.

A major drawback of our paper is the cross-sectional study design because we can confirm group difference only and cannot prove causality. For this reason, the results should be viewed with a certain degree of caution.

Another problem comes from the fact that the data are based on self-reported questionnaires which could have potentially resulted in higher risk of subjective distortions. Further studies should add accelerometers and/or daily logs to optimize measurements and to synchronize available data while evaluating PA in owners of different kinds of animals.

## Conclusion

Physical activity is crucial for maintaining good health in all age groups. Young women who own an animal show significantly higher PA at moderate intensity level. However, important factor of the PA level could be owning a specific kind of animal. This is because exercise habits of horse owners, dog owners and owners of other animals may be different. Important finding of this study is that since physical activity may vary depending on the animal species owned, it is necessary to focus on the diversity of physical activity among owners of specific animal species in future studies. Our study showed that young women who own a horse report higher PA level than those owning a dog or another domestic animal.

## Data Availability

Data will be available on request.

## Abbreviations

AO: animal owners
BMI: Body Mass Index
DO: dog only or dog and other small animal owners /i.e. excluding horses/
HO: horse only or horse plus any other animal owners /i.e. including dogs/
IPAQ: International Physical Activity Questionnaire
MET: metabolic equivalent
MPA: moderate physical activity
NAO: non-animal owners
OAO: all other animal owners, no matter what kind /cats, turtles, snakes, mice, birds/
PA: physical activity
VPA: vigorous physical activity
WHO: World Health Organization
WPA: walking physical activity
